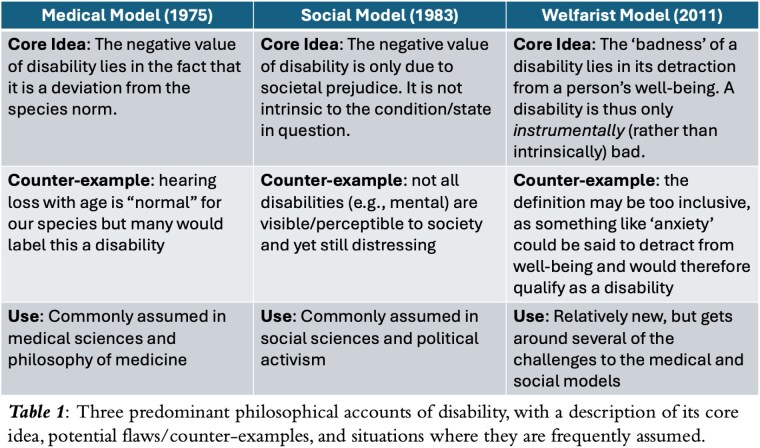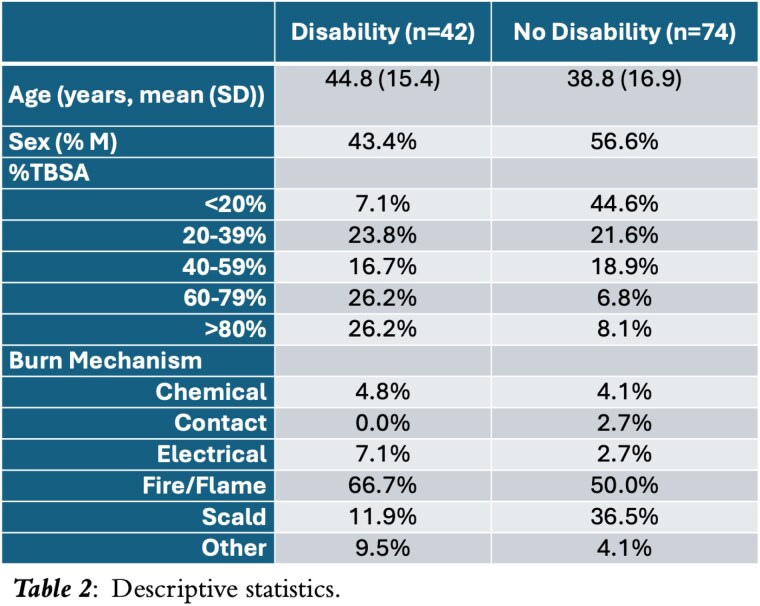# 128 Correlating Self-Reported Disability of Burn Survivors with Philosophical Models of Disability

**DOI:** 10.1093/jbcr/iraf019.128

**Published:** 2025-04-01

**Authors:** Karel-Bart Celie, Eloise Stanton, Cindy Rutter, Daniel Chacon, Maxwell Johnson, Justin Gillenwater, Haig Yenikomshian

**Affiliations:** University of California Keck School of Medicine; University of California Keck School of Medicine; Southern California Burn Model System; Alisa Ann Ruch Burn Foundation; University of California Keck School of Medicine; Los Angeles General Medical Center; University of California Keck School of Medicine

## Abstract

**Introduction:**

Philosophical models provide coherent definitions of concepts that impact how those concepts are subsequently employed in practice. The concept of ‘disability’ has three well-established models, detailed in Table 1. The popular ‘medical’ model would consider all burn survivors disabled, but more contemporary models challenge this. Almost no studies have correlated these models with actual patient experiences.

**Methods:**

A cross-sectional anonymous survey of burn survivors was conducted from January to April of 2023. The association between self-identification of disability and scar visibility, family support, and physical or psychological impact was examined using chi-square and logistic regression analysis. Impact was assessed on a 10-point scale, and an impact of ≥5 was interpreted as significantly detrimental to well-being.

**Results:**

116 responses to questions querying disability were collected. 42 respondents (36%) self-identified as persons with a disability (Table 2). Of those who identified as persons with a disability, 31 (73.8%) indicated having support, compared with 63 (85.1%) who did not identify as having a disability (p=0.135). Patients who identified as having a disability were significantly more likely to report having visible scars (92.9% vs. 70.3%, p=0.004). Multivariate regression analysis demonstrated that when controlling for age, %TBSA, and sex, persons who self-identified as having a disability at 22.1 greater odds of having physical impact detrimental to well-being (OR: 22.1, 95% CI 4.73-103.0, p< 0.001) and 4.7 greater odds of having psychological impact detrimental to well-being (OR: 4.71, 95% CI 1.63-13.6, p=0.004).

**Conclusions:**

Only 36% of respondents identified as having a disability, indicating poor correlation of the medical model with burn survivor experiences. The social model of disability garnered a moderate level of support in this study, since family support did not appear significantly associated with self-identification of disability but having a visible scar did. Finally, given the strong correlation with physical or psychological impact, the welfarist model appears to correlate most strongly with burn survivor self-identification of disability.

**Applicability of Research to Practice:**

This is the first time burn patient self-identification of disability has been correlated with well-established philosophical models. Our findings in the setting of burn survivors suggest that the predominant understanding of disability warrants re-evaluation.

**Funding for the Study:**

The contents of this abstract were developed under a grant from the National Institute on Disability, Independent Living, and Rehabilitation Research (NIDILRR grant number 90DPBU0007). The contents of this abstract do not necessarily represent the policy of NIDILRR, ACL, or HHS, and you should not assume endorsement by the Federal Government.